# Pre- and Postoperative Care for Bariatric Surgery Patients: The Impact of a Designed Reference Guide on Nurses’ Awareness and Patient Satisfaction

**DOI:** 10.3390/healthcare13091023

**Published:** 2025-04-29

**Authors:** Samar Khattab Mohammed, Mahmoud Abdel Hameed Shahin, Fatmah Ahmed Alamoudi, Mosaad M. Morshed, Wafaa Gameel Mohammed Ali, Amal Eid Abdelmonaem Shaaban

**Affiliations:** 1 Faculty of Nursing, Mansoura University, Mansoura 7650030, Egypt; samarkhattab2024@gmail.com; 2 Medical-Surgical and Critical Care Nursing, Nursing Department, Prince Sultan Military College of Health Sciences, Dhahran 34313, Saudi Arabia; 3 Medical-Surgical Nursing, Nursing Department, Prince Sultan Military College of Health Sciences, Dhahran 34313, Saudi Arabia; fatimaamodi@psmchs.edu.sa; 4 General Surgery Department, Faculty of Medicine, Mansoura University, Mansoura 35516, Egypt; morshedmm@hotmail.com; 5 Medical-Surgical Nursing Department, Faculty of Nursing, Mansoura University, Mansoura 7650030, Egypt; denameh@mans.edu.eg (W.G.M.A.); amaleid@mans.edu.eg (A.E.A.S.); 6 Medical-Surgical Nursing Department, Faculty of Nursing, King Khalid University, Khamis Mushait 61421, Saudi Arabia

**Keywords:** nurses’ knowledge, care practice, morbid obesity, perioperative care, obesity surgery

## Abstract

Background/Objectives: Bariatric surgery is a crucial intervention for the treatment of morbid obesity, necessitating that nursing staff possess a thorough understanding of expected outcomes to ensure successful patient care and promote healthier lifestyles. Recognizing this need, the present study aimed to develop and implement a reference guide tailored to nursing staff providing bariatric surgery care. Methods: A quasi-experimental design was employed, utilizing a convenience sample of 78 nurses and 156 patients—comprising 78 participants before the administration of the reference guide and another 78 participants after its provision—from the general surgery units at Mansoura University Hospital. Four tools were employed for data collection: a sociodemographic datasheet, a knowledge questionnaire assessing nurses’ understanding of care for bariatric surgery patients, a self-reported practice questionnaire, and a patients’ satisfaction measurement tool. A reference guide for bariatric surgery care was constructed. Descriptive and inferential statistics were conducted with adherence to all ethical considerations. Results: After implementing the reference guide, the percentage of nurses who exhibited good knowledge of bariatric surgery increased from 10.3% to 80.8%. Additionally, the proportion of nurses reporting poor self-reported practices related to necessary procedures decreased from 80.8% to 5.1%. Surprisingly, the improvement in knowledge and self-reported practices was sustained for a long time, though reduced. Furthermore, patient satisfaction levels showed a marked improvement following the intervention. Conclusion: Implementing a reference guide significantly improved nurses’ knowledge, self-reported practices, and patient satisfaction. To sustain these improvements, it is recommended that the reference guide be made available and educational programs be provided to nursing staff.

## 1. Introduction

Obesity is one of the most significant health issues worldwide and is linked with a higher risk of various diseases including diabetes mellitus type II, cardiovascular conditions, depression, and sleep and respiratory disorders [1,2]. Its prevalence has risen over the past fifty years in both men and women, and despite all efforts to manage it, more than half of the global population is projected to be overweight or to have a degree of obesity by 2030 [3]. The World Health Organization (WHO) reported that Egypt holds the 18th highest prevalence of obesity globally [4]. Given its significant prevalence and detrimental impact on health and healthcare costs, numerous weight loss approaches have been recommended for individuals with obesity [5,6]. Health professionals primarily recommend modifying the diet to focus more on fiber while reducing fat and sugars, along with maintaining a routine of regular exercise and implementing behavioral changes as the core treatments [7,8,9]. Moreover, individuals with obesity can also utilize weight-loss medications that work by suppressing appetite or reducing fat absorption [6]. However, studies indicate that weight regain is probable over time, primarily due to difficulty sustaining new lifestyle changes for an extended period [6,10,11].

Considering the findings of these studies, bariatric surgery should be regarded as a successful option for managing obesity [12], resulting in long-term weight loss and an improvement in specifically related comorbidities [13], which, in turn, increases life expectancy [14]. Gastric bypass and sleeve gastrectomy are the most widely performed bariatric surgery procedures and have been consistently shown to be highly effective in managing obesity. In sleeve gastrectomy, a restrictive technique is employed to reduce the size of the stomach, limiting food intake and promoting a quicker feeling of fullness [15]. In contrast, gastric bypass surgery employs both restrictive and malabsorptive techniques, where bypassing sections of the intestine reduces the absorption of calories and nutrients [16]. Although bariatric surgeries offer numerous benefits, they also carry risks of both immediate and long-term complications [17]. After surgery, patients may encounter complications like bleeding, infections at the surgical site, or problems related to anesthesia. Postoperative immobility can also increase the risk of deep vein thrombosis and pulmonary embolism. Additionally, there is a chance of leakage, which could lead to peritonitis [16,17]. Issues such as dumping syndrome; weight regain; and nutritional deficiencies, particularly in vitamin B12, iron, calcium, and vitamin D, may arise in the long-term postoperatively [16,17]. Therefore, the decision to undergo bariatric surgery should be based on the patient’s individual characteristics and a careful assessment of the potential risks and benefits [18].

Patients scheduled for bariatric surgery must be thoroughly prepared and optimized to minimize risks [19]. The nursing staff plays a crucial role in preparing and supporting these patients throughout the pre- and postoperative period [20]. Nurses can educate patients about the different types of bariatric procedures, expected benefits, long-term outcomes, potential risks, and the importance of lifelong nutritional and behavioral changes, as well as follow-up care to reduce the chance of regaining weight [19,21]. Additionally, nurses ensure patient safety by performing comprehensive health assessments, facilitating clear and effective communication with patients, gathering necessary information, updating the healthcare team on any changes in the patient’s condition, and ensuring the proper implementation of postoperative protocols [19]. Nurses also encourage patients to actively participate in their care, enhancing their quality of life and overall satisfaction throughout the pre- and postoperative period [20].

Donabedian’s Structure–Process–Outcome (SPO) Framework serves as a theory-driven model that logically connects interventions to outcomes [22]. Structure: the reference guide (structural intervention) standardizes knowledge. Process: nurses apply this knowledge in practice (e.g., adherence to protocols). Outcome: improved patient satisfaction due to consistent, evidence-based care. This study is grounded in Donabedian’s SPO framework, where the reference guide (Structure) enhances nurses’ knowledge and adherence to best practices (Process), thereby improving patient satisfaction and clinical outcomes (Outcome). Prior evidence [23,24] confirms that standardized nursing interventions reduce practice variability and elevate patient experiences in postoperative care. Thus, we hypothesize that the guide will function as a mediating tool, translating evidence into consistent practice, ultimately benefiting patients.

According to a study by Aboulghate et al. [4], the prevalence of obesity among adults in Egypt has risen to approximately 40%. Awareness of the long-term risks of obesity and the failure to lose weight through nonsurgical methods often lead individuals with obesity to consider bariatric surgery [12]. Patient care is particularly crucial during the pre- and postoperative phases of bariatric surgery due to several factors, such as the complexity of the procedure, the patient’s condition, potential long-term complications, and their expectations [20]. Nurses are key contributors during this period. Alongside delivering direct care, bariatric nurse practitioners educate patients, monitor their progress post-surgery, modify treatment plans when needed, and coordinate with the bariatric surgery team to ensure that patients are ready for discharge [19]. Given nurses’ vital role, they must use their knowledge to meet patients’ needs. However, the role of nurses is often overlooked in areas such as health education, promotion, counseling, and postoperative follow-up. Some studies have shown that nurses may hold negative attitudes toward overweight patients or patients with obesity and lack sufficient knowledge about obesity. In Egypt, research on nurses’ knowledge and attitudes toward obesity and bariatric surgery is limited. Therefore, it is crucial to address the misconceptions and knowledge gaps in obesity and bariatric surgery among nurses.

This study aimed to develop and implement a comprehensive reference guide for nursing staff involved in pre- and postoperative care for bariatric surgery patients and to evaluate its impact on nurses’ awareness and patient satisfaction levels.

The current study had three research hypotheses:

**H_1_:**  *The implementation of a comprehensive reference guide will significantly improve the nursing staff’s knowledge of pre- and postoperative bariatric care protocols.*

**H_2_:**  *Nurses who are exposed to the reference guide will demonstrate higher confidence in managing bariatric surgery patients compared to those who are not.*

**H_3_:**  *Patients cared for by nurses using the reference guide will report higher satisfaction levels than those cared for by nurses without the guide.*

## 2. Materials and Methods

### 2.1. Research Design

A quasi-experimental research design was utilized to conduct this study. This design is used to estimate the causal impact of implementing the designed reference guide on the target population, “nursing staff”.

### 2.2. Setting

The study was carried out at general surgery units at Mansoura University Hospital from the beginning of September 2023 to the end of May 2024. The units included were 7 and 8 for males and 11 and 12 for females.

### 2.3. Subjects

The study included a convenience sample of 78 nurses from the previously mentioned units who provide direct care for patients with morbid obesity undergoing bariatric surgeries who were willing to participate in the study, were of both sexes, aged 20 to 60 years, and had different qualifications. The study included nearly all nurses working in the specified units during the study period who met the inclusion criteria. Moreover, all patients with morbid obesity admitted during the study period were invited to participate in the research. A total of 78 patients were included before the administration of the reference guide to the nurses, and another 78 patients were included after the provision of the reference guide. The number of bariatric surgery patients was limited; within the 9-month study period, 156 out of 190 patients (82%) participated in the study by agreeing to complete the questionnaire.

### 2.4. Data Collection Tools

Four tools were used for data collection. The researchers developed these tools after reviewing the related old and recent literature, except for the satisfaction scale, which was modified and translated.

Tool I: Sociodemographic data and career characteristics of studied nurses. This included questions about nurses’ age, marital status, level of education, years of experience caring for bariatric surgery patients, previous training, and sources of information.

Tool II: Nurses’ knowledge questionnaire regarding morbid obesity, gastric sleeve, gastric bypass, and pre- and postoperative care for bariatric surgery patients, designed based on literature [25,26,27]. It contained 50 multiple-choice questions covering all bariatric surgery patients from pre-operative preparation before admission until discharge and extended to postoperative instructions.

Tool III: Self-reported practices survey related to nursing procedures designed based on previous studies [28,29,30]. This scale included an assessment of nurses’ knowledge about essential procedures that are carried out for patients pre-and post-bariatric surgery using eight questions related to steps of three main procedures “electrocardiogram, deep breathing and coughing exercise, and pulmonary function test” concerning the main steps and preparation needed (what to do and what to avoid).

Tool IV: Patients’ satisfaction measurement tool, which was modified and translated by Laschinger et al. [31] to assess patients’ satisfaction with nurses’ performance and health education in areas related to routine care, as well as answering patients’ questions related to pre- and postoperative care. It comprises 15 items evaluating: communication (clarity of explanations, preparedness guidance, responsiveness to questions, and family updates), interpersonal care (courtesy, empathy, attentiveness to needs, and respect for preferences), clinical competence (skill in procedures, medication management, and monitoring), and continuity of care (discharge instructions and post-discharge coordination).

Responses are typically scored on a Likert scale of “5 to 1”: (excellent = 5, very good = 4, good = 3, fair = 2, and poor = 1), measuring perceived quality across critical care domains. The items align with Donabedian’s framework, linking nursing processes (e.g., communication) to patient-centered outcomes.

### 2.5. Scoring System of the Tools

The discrete scores for each scale were summed to calculate the total score for each dimension. All scores were then converted to a percentage using the following formula: Score % = (Observed Score/Maximum Score) × 100.

The percentage scores were categorized as follows: for tools II and III, a score of less than 50% was classified as “Poor”, 50% to less than 75% as “Fair”, and 75% or higher as “Good”. For tool IV, the categories were defined as “Dissatisfied” for scores below 50%, “Satisfied” for scores between 50% and less than 75%, and “More satisfied” for scores of 75% or above.

### 2.6. The Reference Guide

The researchers designed a reference guide about pre- and postoperative care for bariatric surgery patients based on reviewing the literature [32,33]. This reference guide aimed to empower nursing staff’s knowledge and practice regarding pre- and postoperative care for patients undergoing sleeve gastrectomy and gastric bypass surgeries.

The reference guide’s content and teaching methods were tailored to nurses’ age, education level, and individual or group needs. The researchers created this guide to present information straightforwardly using simple language and visuals. It was distributed to all nurses promptly to facilitate the reassessment of their knowledge regarding the components of the reference guide.

#### 2.6.1. Validity

Validation was performed for the developed tool and the designed reference guide to determine whether the tool covered the study aim. This stage was carried out by a jury of five experts (two assistant professors from the Faculty of Nursing and three assistant professors from the Faculty of Medicine at Mansoura University). They determined that each item met the criteria for item relevance, achieving an Item-Level Content Validity Index (I-CVI) greater than 0.79. Using a modified Delphi approach, the panel refined the items over two rounds to ensure clarity. Additionally, they confirmed that all key domains were comprehensively covered. The overall scale-level Content Validity Index (S-CVI) was calculated at 0.87, indicating a high level of agreement on the validity of the content.

#### 2.6.2. Reliability

Reliability was assessed using Cronbach’s alpha test. The Arabic version of Tool II demonstrated a reliability score of 0.74, indicating acceptable internal consistency. Tool III exhibited reliability scores of 0.84, which showed good internal consistency across all domains. Similarly, the Arabic version of Tool IV revealed an acceptable reliability and Cronbach’s alpha coefficient of 0.93.

#### 2.6.3. Pilot Study

The pilot study was carried out on 10% of nurses within the selected criteria to test the applicability and relevance of the tool. The data were analyzed, and no radical changes were made in the assessment tools, so nurses who shared in the pilot study were included in the actual study sample.

#### 2.6.4. Procedure

1. Data Collection: Nurses were surveyed to assess their knowledge regarding morbid obesity and bariatric surgery and their ability to care for bariatric surgery patients. Moreover, patients were assessed for satisfaction with nursing care and health education provided to them in the unit.
2. Development and implementation of reference guide: The reference guide was developed by researchers, revised by supervisors, and presented to studied nurses.
3. Implementation: The educational program consisted of six educational sessions delivered to small groups of nurses in the surgical units over nine months, from the beginning of September 2023 to the end of May 2024.
4. Evaluation: An evaluation was conducted to assess the effectiveness of the reference guide on nurses’ knowledge and practice regarding pre- and postoperative care for bariatric surgery patients and patients’ satisfaction levels.

### 2.7. Ethical Considerations

Ethical approval was obtained from the Ethics Committee of the Faculty of Nursing, Mansoura University (ethical approval# 175), and official approval was obtained from the hospital administrative authority. Participants were informed about the study’s nature and purpose, and their participation was voluntary and confidential. Anonymity, privacy, safety, and confidentiality were assured throughout the study, and participants had the right to withdraw at any time. Participants provided informed consent before participating in the study. Overall, these measures ensured the rigor, reliability, and ethical conduct of the study, enhancing its findings’ validity and safeguarding the participants’ rights and well-being.

### 2.8. Statistical Design

After data collection, analysis was conducted using the Statistical Package for the Social Sciences (SPSS) Version 26. Both descriptive and inferential statistics were employed for the analysis. Descriptive statistics included the calculation of means and standard deviations, as well as frequencies and percentages. For inferential statistics, independent sample t-tests and ANOVA were used to identify significant differences in nurses’ knowledge, practices, and patient satisfaction levels across different stages of the study. A *p*-value of less than 0.05 was set to determine statistically significant differences. The correlation coefficient test was also utilized to assess the relationship between nurses’ knowledge and their practices. Finally, the results were presented in tables and figures as appropriate.

## 3. Results

The current study aimed to develop and implement a reference guide for nursing staff involved in the pre- and postoperative care of bariatric surgery patients. The sample consisted of 78 nurses, evaluated for their knowledge and self-reported practices at three intervals: before the implementation, immediately after, and three months following the reference guide’s introduction. Additionally, 78 bariatric surgery patients were assessed regarding their satisfaction with the pre- and postoperative nursing care and health education they received, both before and after implementing the reference guide for nurses.

Regarding the sociodemographic characteristics of the nursing staff in Table 1, a significant portion of the nurses, 47.4%, were under 30, indicating a relatively young workforce. This was followed by 32.1% of nurses aged between 30 and 39 years, while those aged 40 to 59 comprised 20.5% of the sample.

Regarding gender, the nursing staff was predominantly female, with 94.9% identifying as women, which reflects a common trend in the nursing profession. Only 5.1% of the nurses were male. Marital status among the nurses showed that the majority were married, accounting for 62.8% of the sample. The remaining nurses were divided among those who were divorced (12.8%), widowed (19.2%), and single (5.1%).

The educational background of the nursing staff varied, with most nurses (55.1%) having completed secondary nursing school. Additionally, 34.6% held diplomas from the higher institute of nursing, while only 10.3% obtained bachelor’s degrees. This distribution suggested a need for ongoing education and professional development within the nursing team. Regarding residence, 52.6% of the nurses lived in rural areas, while 47.4% resided in urban settings, highlighting a nearly even split between the two environments.

Finally, income levels revealed that a vast majority of the nurses (94.9%) reported having sufficient income, while a small fraction (5.1%) indicated that their financial situation was insufficient.

As for the career characteristics of the nursing staff, a significant majority of nurses (83.3%) had six or more years of experience in surgical settings. This indicated a well-established workforce with substantial expertise in surgical care. Furthermore, regarding their experience with patients of morbid obesity undergoing bariatric surgery, an impressive 94.9% reported having cared for such patients six times or more, demonstrating a strong familiarity with this specific patient population.

Regarding professional development, the data showed that 52.6% of the nurses had not attended any training courses, while 47.4% had participated in previous training. Of those who attended those training courses, only 27% reported that the courses were related to obesity and bariatric surgery, while the rest (73%) attended other specialty courses. Regarding sources of information about obesity and bariatric surgery, the nurses predominantly relied on television programs, with 80.8% citing this as their primary source. The internet was used by 19.2% of the respondents, highlighting a preference for traditional media over digital resources for obtaining information.

In assessing the sufficiency of information available to patients about obesity, 70.5% of nurses believed the information provided was nearly adequate. However, 10.3% felt it was insufficient, while 15.4% considered it limited. Only a small percentage (3.8%) reported that the information was abundant and comprehensive.

The methods employed by nurses to convey necessary information to patients primarily included interviews and oral explanations (55.1%) and telephone calls (43.6%). In terms of essential information that should be communicated to patients, an overwhelming 92.3% of nurses indicated that preparation for surgery was crucial. In contrast, only 6.4% believed postoperative complications should be included, and just 1.3% emphasized the importance of lifestyle modifications after surgery.

Overall, these findings highlighted the nursing staff’s extensive experience and reliance on traditional media for information related to obesity and bariatric surgery. They also pointed to potential gaps in patient education and underscored the need for focused efforts to enhance the delivery of pre- and postoperative care for bariatric surgery patients (Table 1).

The findings regarding nurses’ knowledge of pre- and postoperative care for bariatric surgery patients, as presented in Table 2, illustrate the impact of the reference guide at three critical time points: pre-implementation, post-implementation, and follow-up. Before implementing the reference guide, the mean total knowledge score among the nursing staff was 15.92 (±4.69) on a scale of 0 to 50. This was translated to a normalized mean score of 0.32 (±0.09), indicating a low level of knowledge regarding the specific needs of bariatric surgery patients during the pre- and postoperative period.

After introducing the reference guide, there was a notable increase in the nurses’ knowledge. The mean total knowledge score rose significantly to 41.08 (±5.27), resulting in a normalized mean score of 0.82 (±0.11). This improvement suggested that the reference guide effectively enhanced the nurses’ understanding and preparedness to care for bariatric surgery patients.

However, during the follow-up assessment, the mean total knowledge score decreased to 35.40 (±5.80), corresponding to a normalized mean score of 0.71 (±0.12). While this score remained higher than the pre-implementation level, it indicated a decline in knowledge retention over time since the initial post-implementation measurement. Overall, these findings highlighted the positive impact of the reference guide on nurses’ knowledge in the short term while also emphasizing the importance of ongoing education and reinforcement to maintain this knowledge in the long run.

Using ANOVA Scheffe post hoc tests to analyze the impact of the designed reference guide on nurses’ knowledge of pre- and postoperative care for bariatric surgery patients across three phases, the analysis revealed significant mean differences among the groups. Notably, the mean knowledge score of nurses increased substantially from the pre-implementation phase to the post-implementation phase, with a mean difference of −25.15 (*p* < 0.001). This indicates a significant improvement in nurses’ knowledge following the implementation of the reference guide.

When comparing the post-implementation phase to the follow-up phase, the mean difference was 5.68 (*p* < 0.001), suggesting a notable decline in knowledge retention over time. Additionally, the follow-up group displayed a significant mean difference of 19.47 compared to the pre-implementation group (*p* < 0.001). This finding highlighted that although knowledge decreased from post-implementation to follow-up, it remained significantly higher than the pre-implementation level.

Overall, these findings demonstrate that the implementation of the reference guide substantially enhanced nurses’ knowledge of pre- and postoperative care for bariatric surgery patients, although a decline in knowledge retention was observed at the follow-up stage.

**Figure 1:**
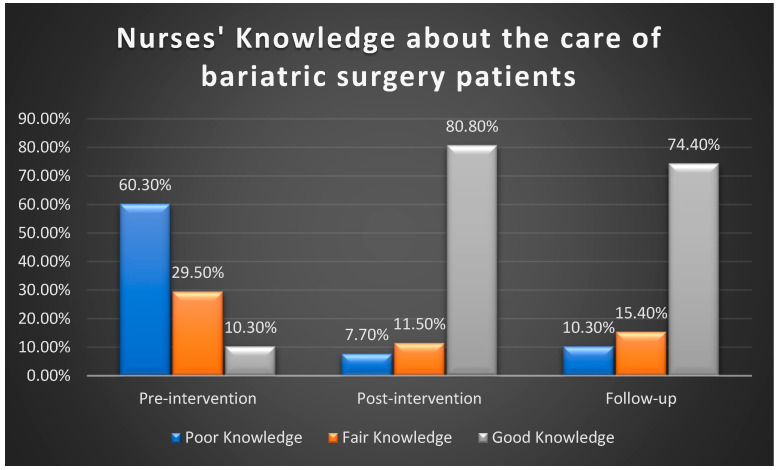
Comparison of total nurses’ knowledge about the care of bariatric surgery patients.

Utilizing total knowledge cut scores to classify nurses into three categories—poor, fair, and good knowledge—Figure 1 illustrates remarkable changes in knowledge levels across the study phases. In the pre-intervention phase, 60.3% of the nurses had poor knowledge. This proportion decreased substantially to 7.7% in the post-intervention phase and 10.3% during the follow-up phase. Conversely, the percentage of nurses with good knowledge increased notably from 10.3% in the pre-intervention phase to 80.8% post-intervention and then slightly declined to 74.4% in the follow-up phase.

The findings regarding nurses’ self-reported practices in the pre- and postoperative care of bariatric surgery patients, as outlined in Table 3, revealed significant changes across three key time points: pre-implementation, post-implementation, and follow-up.

Before implementing the reference guide, the mean total self-reported practices score was 2.92 (±1.29) on a scale from 0 to 8. This was translated to a normalized mean score of 0.37 (±0.16), indicating that nurses had a relatively low level of self-reported practices in the care of bariatric surgery patients.

Following the introduction of the reference guide, there was a marked improvement in these practices. The mean total self-reported practices score rose to 6.22 (±1.25), resulting in a normalized mean score of 0.78 (±0.16). This increase highlights the positive impact of the reference guide on enhancing nurses’ practices in pre- and postoperative care.

However, during the follow-up assessment, the mean total self-reported practices score dropped to 5.01 (±1.16), corresponding to a normalized mean score of 0.63 (±0.15). While this score reflected a decrease from the post-implementation level, it remained higher than the pre-implementation score, indicating that the improvements in practices were partially retained over time. These findings demonstrated that implementing the reference guide enhanced nurses’ self-reported practices effectively in the pre- and postoperative care of bariatric surgery patients, although some decline in retention was observed in the follow-up stage.

Using the ANOVA Scheffe post hoc test to analyze the impact of the designed reference guide on nurses’ self-reported practices in the pre- and postoperative care of bariatric surgery patients, the analysis revealed significant mean differences between the groups. From the pre-implementation phase to the post-implementation phase, the mean difference was −3.29 (*p* < 0.001), indicating a substantial improvement in self-reported practices following the implementation of the reference guide.

In comparing the post-implementation phase to the follow-up phase, the mean difference was 1.21 (*p* < 0.001), which indicated a significant decline in practices in the follow-up period. Additionally, the follow-up group showed a significant mean difference of 2.09 compared to the pre-implementation group (*p* < 0.001), which underscores that even after the follow-up period, nurses maintained a significantly higher level of self-reported practices compared to their initial scores.

Overall, the findings demonstrated that implementing the reference guide significantly enhanced nurses’ self-reported practices in the pre- and postoperative care of bariatric surgery patients. However, a decline was noted in the practice scores at the follow-up stage.

**Figure 2:**
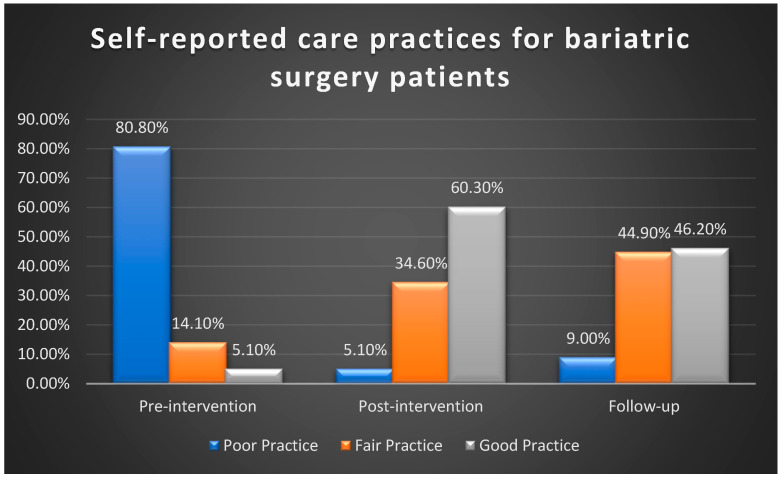
Comparison of total nurses’ self-reported practices regarding pre- and postoperative procedures (n = 78).

Using total self-reported practice cut scores to classify nurses into three categories—poor, fair, and good practice—Figure 2 illustrates that nurses’ self-reported practices regarding necessary procedures and pre- and postoperative care for bariatric surgery patients improved following the intervention. Before implementing the reference guide, 80.8% of nurses reported poor practices. This figure decreased significantly to 5.1% post-implementation and 9% at the follow-up. Additionally, only 5.1% of nurses reported good self-reported practice before the intervention, which increased remarkably to 60.2% after the implementation but decreased to 46.2% in the follow-up stage measurement.

Table 4 details the patient satisfaction levels concerning pre- and postoperative care and health education provided by nurses, comparing scores before and after implementing the reference guide for bariatric surgery patients.

The study included 78 patients in both the pre- and post-implementation groups. Before the reference guide implementation, the average satisfaction score was 2.65 (±0.223) on a scale of 1 to 5. The mean satisfaction score after the implementation increased to 3.06 (±0.281).

The Independent Samples t-test revealed a statistically significant difference between the two groups, with a *p*-value < 0.000. This result indicated that implementing the reference guide significantly enhanced bariatric surgery patient satisfaction with the pre- and postoperative nursing care and health education offered in the surgical units.

**Figure 3:**
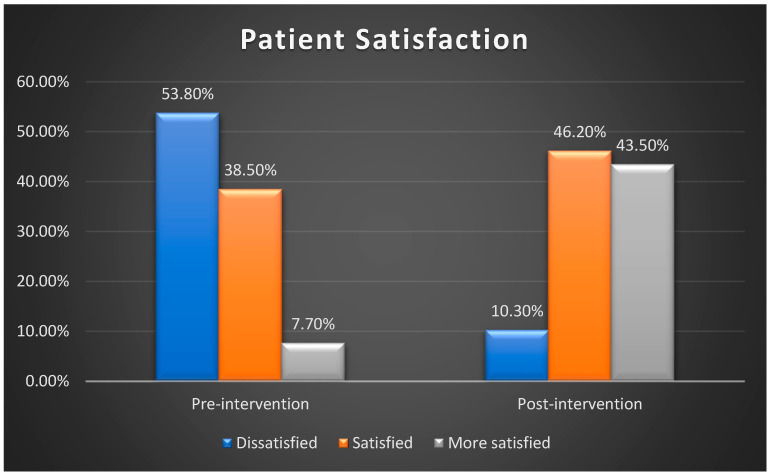
Comparison of the patients’ satisfaction level pre- and post-intervention (n = 78).

Using total patient satisfaction cut scores to classify responses into three categories—dissatisfied, satisfied, and more satisfied—Figure 3 illustrates a notable increase in patient satisfaction levels following the implementation of the reference guide. Before the intervention, 53.8% of patients reported dissatisfaction, which decreased to 10.3% post-implementation. Additionally, the percentage of patients in the more satisfied category rose from 7.7% in the pre-intervention phase to 43.5% after the intervention.

Table 5 represents the correlation between nurses’ knowledge and practices in the pre- and postoperative care of bariatric surgery patients. The Pearson correlation coefficient indicated a strong positive relationship between total knowledge and self-reported practice, with a correlation value of 0.784 (*p* < 0.01). This significant finding suggested that higher levels of knowledge among nurses were associated with improved self-reported practices in the pre- and postoperative care of bariatric surgery patients. The statistical significance of this correlation underscores the importance of enhancing nurses’ knowledge to positively influence their care practices.

## 4. Discussion

The current study aimed to develop and implement a reference guide specifically designed for nursing staff concerning pre- and postoperative care for bariatric surgery patients. We evaluated the impact of this reference guide on nurses’ knowledge and practice in pre- and postoperative care for bariatric surgery patients. Additionally, we examined the relationship between patients’ satisfaction with pre- and postoperative care and the health education provided by nurses.

Regarding the attendance of training courses related to obesity and bariatric surgery, only one-quarter (27%) of the nurses who attended previous educational courses reported that they had participated in specialized bariatric surgery courses during their tenure. Additionally, nurses indicated that their primary sources of information about obesity and bariatric surgery were TV programs and the internet. This finding may stem from the insufficient availability of hospital-based programs or conferences that specifically focus on the responsibilities of nurses in bariatric surgery. Our finding aligns with a study conducted by Aziz and Mohammed [34], which indicated that 84% of the nurses in their sample did not take part in national training sessions focused on bariatric surgery. The authors attributed this outcome to the regular rotation of nurses out of the surgical wards, which may adversely affect the quality of care given to patients undergoing bariatric surgery. Furthermore, our findings align with a study by Fan et al. [35], where nurses indicated that they primarily acquired information about bariatric surgery from mass media sources like TV and websites. Only a small proportion of nurses referred to school education (5.6%) and government education (4.9%) as their sources of knowledge [35].

The current study revealed a low level of knowledge among nurses caring for bariatric surgery patients regarding their specific needs during pre- and postoperative care before implementing the reference guide. This knowledge gap included a limited understanding of obesity, its causes, body mass index classifications, and the different types and indications of bariatric surgeries. Moreover, nurses had insufficient awareness of the risks associated with the surgery, the needs of the patients, and their own responsibilities, such as offering education and discharge guidance to help prevent complications and weight regain throughout the pre- and postoperative phases. The lack of knowledge among nurses in this area might result from the absence of educational training programs, resources, and materials focused on obesity care. This may also be influenced by the educational levels of the participating nurses, with only 10% holding a bachelor’s degree, while the majority had diplomas from high nursing institutes or secondary nursing schools. Consequently, it is likely that the nursing curriculum’s coverage of obesity-related topics is relatively limited.

This result is consistent with a study by Mansour et al. [25] about nurses’ performance for patients undergoing bariatric surgery, which emphasized that nurses had inadequate knowledge about obesity as a chronic disease and how to manage bariatric surgery patients. Our findings align with a study by Fan et al. [35], which identified many deficiencies and misunderstandings about obesity among Chinese nurses. For instance, the study revealed a lack of knowledge among participants regarding obesity-related disorders, such as cancer, gastroesophageal reflux disease, and psychological disorders. Additionally, the Chinese nurses were unable to correctly classify their own weight based on BMI, as most of them believed they were overweight, while they were of a normal, healthy weight [35]. The authors linked this result to the high personal and societal expectations of ideal body weight among Chinese nurses. Thus, the authors suggested that nurses should be proficient in using BMI, waist circumference, hip circumference, and other important measures to assess obesity [35].

Our results also agreed with other studies that assessed nurses’ knowledge about postoperative care following bariatric surgery [25,27,36]. The results of the current study contrasted with those of Moyo, Felix [37], who indicated that participants had above-average knowledge of obesity. This discrepancy could be because half of the nurses in that study possessed a bachelor’s degree or higher and cared for more patients with obesity as they worked in private healthcare settings [37]. Following the implementation of the reference guide, there was a significant increase in the average level of nurses’ knowledge about bariatric surgery compared to the pre-implementation phase, demonstrating a marked improvement in their comprehension. These results are consistent with the findings of Aziz, Mohammed [34], who demonstrated a statistically significant increase in nurses’ knowledge about the nursing management of pre- and post-bariatric surgery when evaluating the effectiveness of a nursing instructional intervention program [34].

In terms of nursing knowledge related to specific pre- and postoperative care practices for bariatric surgery patients, including using electrocardiograms (ECG), performing deep breathing exercises, and conducting pulmonary function tests, the study found that participating nurses had a low level of knowledge before the reference guide was implemented. After implementing the reference guide, nurses’ self-reported knowledge was improved. The findings align with Elawadi et al. [38], who reported a significant increase in nurses’ knowledge about ECG following the implementation of an educational intervention designed to assess the effectiveness of the training on ECG procedures. Our result is consistent with the findings of Mahran, Radwan [39], who observed that the average knowledge score of internship nurses in the intensive care unit regarding breathing and coughing exercises significantly improved after they participated in an educational program focused on managing patients with acute exacerbation of Chronic Obstructive Pulmonary Disease. Before the program, the nurses had low knowledge scores related to deep breathing exercises.

Additionally, our findings are consistent with those of Halemaneyavaradananagowda, Gowda [40], who stated that nearly half of their participants demonstrated insufficient knowledge regarding pulmonary function tests, including their indications and procedures. It was noted that nurses’ knowledge of morbid obesity, bariatric surgeries, and pre- and postoperative care practices for bariatric surgery patients declined during the follow-up phase compared to immediately after the reference guide was implemented, though it remained higher than before the guide was introduced. Moreover, a notable connection was observed between nurses’ knowledge and their practices in the pre- and postoperative care of bariatric surgery patients. This emphasizes the necessity of enhancing nurses’ knowledge to positively affect their pre- and postoperative care practices. These findings collectively highlight the need for nurses to update their knowledge and practices in bariatric surgery. It is essential for nurses working in this field to participate in continuous education through relevant sessions to stay current.

In terms of patient satisfaction, our study revealed a significant improvement in satisfaction levels related to pre- and postoperative care and the health education provided by nurses after the reference guide was applied. This finding is consistent with the results of Cheng et al. [41] and Abd El-Naby, Elmetwaly [42], which both reported moderate levels of patient satisfaction with nursing management and education. Patients in these studies noted that the services, including the education provided by nurses, contributed to a reduction in complications associated with bariatric surgery and enhanced their quality of life following the procedure [41,42]. While our findings demonstrate significant improvements in patient satisfaction across communication, interpersonal care, and discharge preparedness, we recognize that linking these results to objective clinical outcomes (e.g., 30-day readmissions, weight loss maintenance, postoperative complications, and lifestyle adherence) would strengthen the evidence. Patient satisfaction remains a validated proxy for care quality in surgical settings [43,44], but future iterations of this work should integrate electronic health record data or follow-up surveys to assess downstream effects.

The clinical implications of the study encompass several key areas that could significantly enhance patient care. By providing evidence for improved care and treatment protocols for pre- and postoperative care of patients with bariatric surgeries, this study may lead to better health outcomes for patients. Additionally, the findings could inform clinical practice guidelines, helping nurses and healthcare professionals make more informed decisions. The research may also identify gaps in provider knowledge, prompting improvements in training and continuing education. Furthermore, the results could support necessary policy changes and optimize resource allocation, ultimately fostering a safer and more collaborative healthcare environment. Overall, the study’s insights have the potential to drive ongoing improvements in clinical nursing practices and patient care.

This research has several limitations. For example, it relied on self-reported practices rather than directly assessing skills. Additionally, data were collected from a single hospital in Egypt, which may restrict the generalizability of the findings. The homogeneity of our sample may reduce variability and limit generalizability; therefore, future studies with larger samples are needed to facilitate more sophisticated statistical adjustments while continuing to prioritize real-world clinical implementation. Additionally, while this study provides valuable insights into patient satisfaction with pre- and postoperative nursing care, the findings may have cultural and contextual limitations that could affect generalizability to other healthcare settings or patient populations.

## 5. Conclusions

This study aimed to develop and implement a reference guide for nursing staff involved in the pre- and postoperative care of bariatric surgery patients. Additionally, it sought to evaluate the effectiveness of the reference guide on nurses’ knowledge and practices regarding pre- and postoperative care for bariatric surgery patients, as well as patients’ satisfaction levels. Nursing knowledge about obesity, bariatric surgeries, and associated complications, along with the necessary pre- and postoperative practices, significantly improved after the guide’s implementation. This improvement contributed to increased patient satisfaction with nursing management, including patient education. Overall, this study emphasized the importance of conducting continuous educational sessions and conferences for nurses who care for bariatric surgery patients to enhance their awareness and keep them updated with the latest developments in the field.

Recommendations:

It is recommended that the level of nursing knowledge regarding the care of bariatric surgery patients be continuously assessed. Strategies such as attending training courses and conferences can help enhance nurses’ knowledge. Barriers that affect the nurses’ knowledge need to be monitored. Updating the nursing curriculum with content related to obesity is also important for increasing nurses’ knowledge. Moreover, it is recommended to provide a reference guide, including an Arabic version, to all nursing staff providing direct care to bariatric surgery patients to ensure they have all the necessary information. Finally, while assessing patient satisfaction before and after reference guide implementation yields important insights, future research should expand evaluation to include postoperative complications, lifestyle adherence, and long-term health outcomes for a more complete assessment of the intervention’s effectiveness.

## Figures and Tables

**Table 1:** Nurses’ distribution according to their sociodemographic and career data (n = 78).

Sociodemographic Variables		n	%
Age	20 < 30 years	37	47.4
	30 < 39 years	25	32.1
	40 < 60 years	16	20.5
Gender	Male	4	5.1
	Female	74	94.9
Marital Status	Single	4	5.1
	Married	49	62.8
	Divorced	10	12.8
	Widow	15	19.2
Educational Level	Bachelor’s degree	8	10.3
	Higher Institute of Nursing	27	34.6
	Secondary Nursing School	43	55.1
Residence	Rural	41	52.6
	Urban	37	47.4
Income	Insufficient	4	5.1
	Sufficient	74	94.9
Work in surgery (Years)	1 < 6 years	13	16.7
	6 years or more	65	83.3
How many times did you care for patients with morbid obesity undergoing bariatric surgery?	4–5 times	4	5.1
	6 times or more	74	94.9
Have you attended previous training courses?	No	41	52.6
	Yes	37	47.4
If yes, what were the courses about? (n = 37)	Obesity and bariatric surgeries	10	27
	Others	27	73
What is your source of information related to obesity and bariatric surgery?	TV programs	63	80.8
	Internet	15	19.2
Information about obesity	Not enough for patients	8	10.3
	Little and try to increase it	12	15.4
	Nearly enough	55	70.5
	A lot and covers obesity and bariatric surgery	3	3.8
What are the ways that you use to provide patients with needed information?	Telephone calls	34	43.6
	Interviews and oral explanation	43	55.1
	Others	1	1.3
Information given to the patient must include	Preparation for surgery	72	92.3
	Complications after surgery	5	6.4
	Lifestyle modification after surgery	1	1.3

**Table 2:** Impact of implementing the designed reference guide on nurses’ knowledge regarding the care of bariatric surgery patients.

Measurement Stage		Total Knowledge Score (0–50)		Mean of Total Knowledge (0–1)	
		Mean ± SD		Mean ± SD	
Pre-implementation		15.92 ± 4.69		0.32 ± 0.09	
Post-implementation		41.08 ± 5.27		0.82 ± 0.11	
Follow-up		35.40 ± 5.80		0.71 ± 0.12	
(I) Group		Mean Difference (I-J)	Sig.	95% Confidence Interval	
				Lower Bound	Upper Bound
Pre-implementation	Post-implementation	−25.15385 *	0.000	−27.2342	−23.0735
	Follow-up	−19.47436 *	0.000	−21.5547	−17.3940
Post-implementation	Pre-implementation	25.15385 *	0.000	23.0735	27.2342
	Follow-up	5.67949 *	0.000	3.5991	7.7599
Follow-up	Pre-implementation	19.47436 *	0.000	17.3940	21.5547
	Post-implementation	−5.67949 *	0.000	−7.7599	−3.5991

* The mean difference is significant at the 0.05 level. Dependent variable: total knowledge score.

**Table 3:** Impact of implementing the designed reference guide on nurses’ practices of pre- and postoperative care of bariatric surgery patients.

Measurement Stage		Total Self-Reported Practices Score (0–8)		Mean of Total Self-Reported Practices (0–1)	
		Mean ± SD		Mean ± SD	
Pre-implementation		2.92 ± 1.29		0.37 ± 0.16	
Post-implementation		6.22 ± 1.25		0.78 ± 0.16	
Follow-up		5.013 ± 1.16		0.63 ± 0.15	
(I) Group		Mean Difference (I-J)	Sig.	95% Confidence Interval	
				Lower Bound	Upper Bound
Pre-implementation	Post-implementation	−3.29487 *	0.000	−3.7804	−2.8094
	Follow-up	−2.08974 *	0.000	−2.5752	−1.6043
Post-implementation	Pre-implementation	3.29487 *	0.000	2.8094	3.7804
	Follow-up	1.20513 *	0.000	0.7196	1.6906
Follow-up	Pre-implementation	2.08974 *	0.000	1.6043	2.5752
	Post-implementation	−1.20513 *	0.000	−1.6906	−0.7196

* The mean difference is significant at the 0.05 level. Dependent Variable: total self-reported practices.

**Table 4:** Bariatric surgery patients’ satisfaction with pre- and postoperative care and nurses’ health education.

Measurement Stage		N	Mean	Std. Deviation	T-Test
Average total patient satisfaction	Pre-implementation	78	2.65	0.223	*p* < 0.000 *
	Post-implementation	78	3.06	0.281	

* The mean difference is significant at the 0.05 level.

**Table 5:** Correlation between nurses’ knowledge and practices of pre- and postoperative care of bariatric surgery patients.

Correlations		Total Knowledge	Total Self-Reported Practice
Total knowledge	Pearson Correlation	1	0.784 **
	Sig. (2-tailed)		0.000
	N	234	234
Total self-reported practice	Pearson Correlation	0.784 **	1
	Sig. (2-tailed)	0.000	
	N	234	234

** Correlation is significant at the 0.01 level (2-tailed).

## Data Availability

All data related to the current research are expressed in this article.

## Abbreviations

WHO	World Health Organization.
SPO	Donabedian’s Structure–Process–Outcome.
